# A deep learning-based electrocardiogram risk score for long term cardiovascular death and disease

**DOI:** 10.1038/s41746-023-00916-6

**Published:** 2023-09-12

**Authors:** J. Weston Hughes, James Tooley, Jessica Torres Soto, Anna Ostropolets, Tim Poterucha, Matthew Kai Christensen, Neal Yuan, Ben Ehlert, Dhamanpreet Kaur, Guson Kang, Albert Rogers, Sanjiv Narayan, Pierre Elias, David Ouyang, Euan Ashley, James Zou, Marco V. Perez

**Affiliations:** 1 Department of Computer Science, Stanford University, Palo Alto, CA USA; 2 Department of Medicine, Stanford University, Palo Alto, CA USA; 3 Department of Biomedical Informatics, Stanford University, Palo Alto, CA USA; 4 Department of Biomedical Informatics, Columbia University Irving Medical Center, New York, NY USA; 5 Milstein Division of Cardiology, Department of Medicine, Columbia University Irving Medical Center, New York, NY USA; 6 Department of Cardiology, Smidt Heart Institute, Cedars-Sinai Medical Center, Los Angeles, CA USA; 7 Department of Biomedical Data Science, Stanford University, Palo Alto, CA USA

**Keywords:** Risk factors, Software, Machine learning

## Abstract

The electrocardiogram (ECG) is the most frequently performed cardiovascular diagnostic test, but it is unclear how much information resting ECGs contain about long term cardiovascular risk. Here we report that a deep convolutional neural network can accurately predict the long-term risk of cardiovascular mortality and disease based on a resting ECG alone. Using a large dataset of resting 12-lead ECGs collected at Stanford University Medical Center, we developed SEER, the Stanford Estimator of Electrocardiogram Risk. SEER predicts 5-year cardiovascular mortality with an area under the receiver operator characteristic curve (AUC) of 0.83 in a held-out test set at Stanford, and with AUCs of 0.78 and 0.83 respectively when independently evaluated at Cedars-Sinai Medical Center and Columbia University Irving Medical Center. SEER predicts 5-year atherosclerotic disease (ASCVD) with an AUC of 0.67, similar to the Pooled Cohort Equations for ASCVD Risk, while being only modestly correlated. When used in conjunction with the Pooled Cohort Equations, SEER accurately reclassified 16% of patients from low to moderate risk, uncovering a group with an actual average 9.9% 10-year ASCVD risk who would not have otherwise been indicated for statin therapy. SEER can also predict several other cardiovascular conditions such as heart failure and atrial fibrillation. Using only lead I of the ECG it predicts 5-year cardiovascular mortality with an AUC of 0.80. SEER, used alongside the Pooled Cohort Equations and other risk tools, can substantially improve cardiovascular risk stratification and aid in medical decision making.

## Introduction

Cardiovascular disease is the most common cause of death in the United States and globally despite the availability of preventive therapies^1^. Prescription of these therapies relies on allocating preventative care to higher-risk patients, making accurate risk stratification invaluable^2^. Even so, commonly used risk scores like the pooled cohort equations (PCE) for risk stratification of atherosclerotic disease^3^ (ASCVD) suffer from limited accuracy and leverage only a few simple risk factors including cholesterol, blood pressure, and age^4^, despite the range of rich data sources available.

Proposed improvements to the PCE require additional data from imaging or lab testing that can be both extensive and beyond what is currently included in standard of care management. In cases of intermediate PCE risk, measuring coronary artery calcium (CAC) further stratifies patients but incurs the additional cost of computed tomography imaging^5^. Polygenic risk scores can identify patients with greater genetic risk for cardiovascular disease^6^ but require genetic sequencing and fail to account for a lifetime of environmental risk. Risk scores can be developed based on larger collections of multimodal features to improve accuracy^7^, but implementation is difficult since measurements often are missing in practice. A risk score that can further risk stratify those at low or intermediate ASCVD risk from existing or inexpensive and easily-acquired data would fill a gap in clinical practice and provide significant additional value.

Discrete abnormalities in the electrocardiogram (ECG), including signs of left ventricular hypertrophy^8^, bundle branch blocks^9^, and premature ventricular contractions^10^, are individually associated with modestly increased cardiovascular and all-cause mortality^11^ and with higher incidence of major cardiovascular events^12^. Given its low cost and near-ubiquity, the ECG is a good candidate for risk scoring. Still, there has been limited success in using the ECG to assess cardiovascular risk in the general population^13^. Convolutional Neural Networks (CNNs) trained on large datasets can learn clinically relevant patterns in raw ECG waveforms, often matching or surpassing cardiologist performance on tasks ranging from standard interpretation^14^ to diagnosis of diseases like cardiac contractile dysfunction^15^, hypertrophic cardiomyopathy versus hypertension^16^, and atrial fibrillation in patients in sinus rhythm^17^. Most relevant to our work, CNNs are able to predict short-term all-cause^18^ and post-operative^19^ mortality with high accuracy based on ECG alone. Predicting long-term cardiovascular mortality, on the other hand, has not been previously explored but has large implications for clinical intervention such as decisions about statin use.

Using a large dataset of ECGs from Stanford University Medical Center (Stanford), we developed SEER, the Stanford Estimator of ECG Risk, a CNN-based risk score to predict long-term risk of cardiovascular mortality and other cardiovascular diseases from only a single resting 12-lead ECG. SEER is trained on Stanford ECGs to predict 5-year cardiovascular mortality, but can accurately predict a range of cardiovascular disease across an array of time-scales. It stratifies patients in five different evaluation cohorts from three different medical centers and two different ECG vendors, and out-performs predictions based on traditional risk factors. When used together with the PCE, SEER reclassifies patients to better predict cardiovascular disease and mortality. We envision SEER being used alongside the PCE score to evaluate cardiovascular risk in ambulatory settings including in outpatient clinics and on wearable devices.

## Results

### Study populations

SEER was trained using a dataset of resting ECGs from Stanford University Medical Center (Stanford; Supplementary Table 1, Supplementary Fig. 1) and evaluated using ECGs from Stanford, Cedars-Sinai Medical Center (Cedars-Sinai; Supplementary Table 2, Supplementary Fig. 2), and Columbia University Irving Medical Center (Columbia; Supplementary Table 3). A set of 312,422 Stanford ECGs with either a cardiovascular mortality within 5 years or 5 years of followup was used to train SEER to predict 5-year cardiovascular mortality. SEER was evaluated on three held-out test sets, the Stanford, Cedars-Sinai, and Columbia test sets of 31,899, 46,095, and 458,455 patients respectively, each consisting of first available ECGs per patient. Additionally, we report results on the Stanford PCE comparison set, a subset of the Stanford cross-validation set with 18,370 ECGs, and the Cedars-Sinai PCE comparison set, a subset of the Cedars-Sinai test set with 4065 ECGs, two datasets of healthy patients with associated clinical data.

### SEER accurately predicts cardiovascular mortality and ASCVD events across three sites

**Fig. 1:**
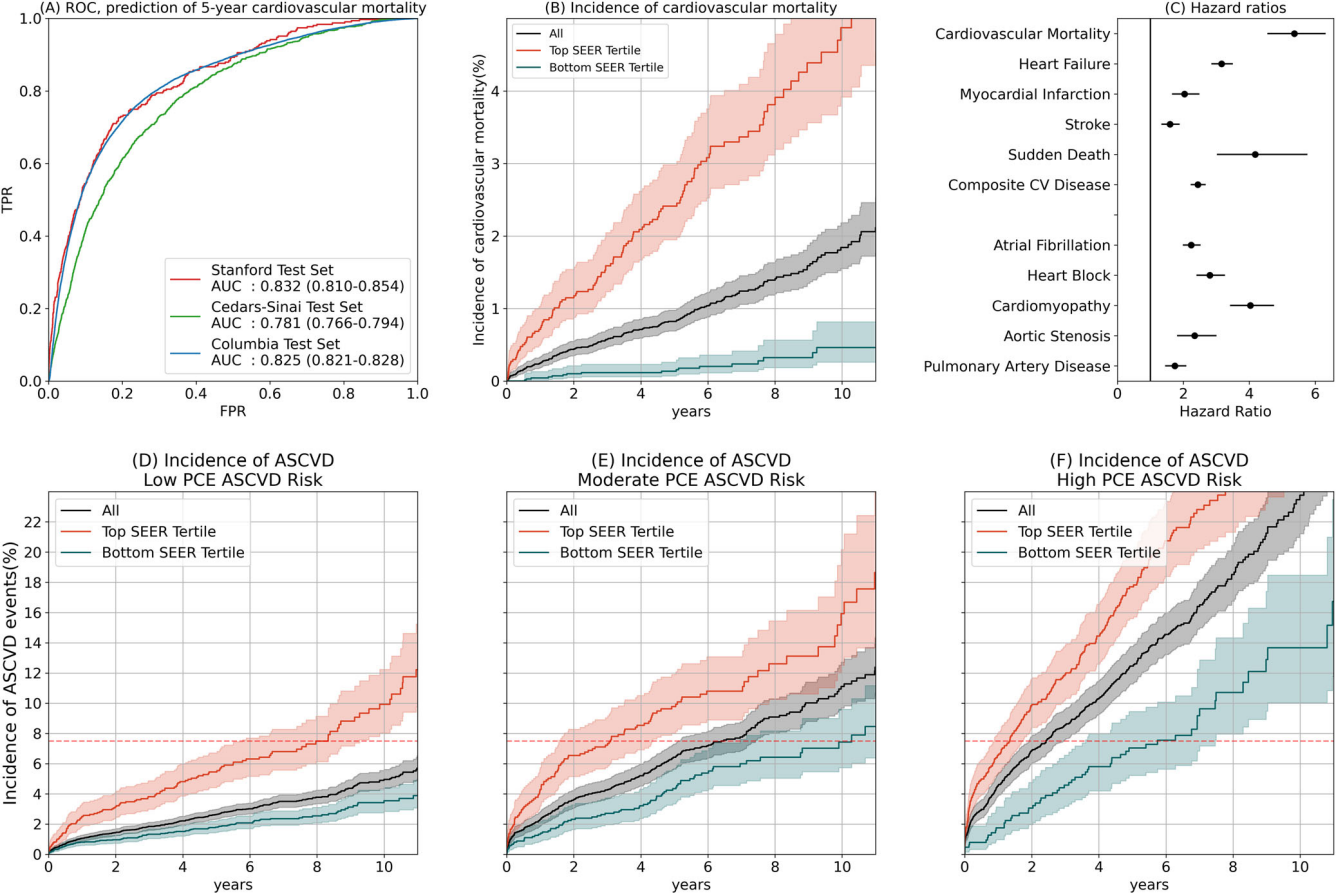
Performance of the SEER Model. **A** Receiver Operator Characteristic (ROC) curves and Areas Under the Curve (AUCs) for Stanford, Cedars-Sinai, and Columbia test sets. **B** Cumulative incidence of cardiovascular mortality in the Stanford PCE comparison set (Kaplan-Meier estimates). The blue and red lines represent the bottom and top third of patients as ranked by SEER; the black line represents all patients. **C** Hazard ratios of various cardiovascular diseases given that a patient is in the top tertile of SEER risk in the Stanford test set. **D**–**F** Cumulative incidence of atherosclerotic cardiovascular disease in the Stanford PCE comparison set (Kaplan–Meier estimates), among patients called low-risk (0–7.5%) moderate risk (7.5%–20%), and high-risk (20–100%) by the PCE. The blue and red lines represent the bottom and top tertiles of patients as ranked by SEER; the black line represents all patients. The dotted red line shows the 7.5% risk cutoff used in the decision to prescribe statins. All error bars represent 95% bootstrap confidence intervals.

We first investigated SEER’s performance in predicting cardiovascular mortality based on a single 12-lead ECG in the three test sets (Table 1). We defined cardiovascular mortality as a mortality falling within thirty days of a myocardial infarction, ischemic stroke, intracranial hemorrhage, sudden cardiac death, or hospitalization for heart failure. Among patients in these cohorts with 5 years of followup or a cardiovascular mortality within 5 years, SEER predicted 5-year cardiovascular mortality with areas under the receiver operator characteristic curve (AUC) of 0.83 (95% CI: 0.81–0.85), 0.78 (0.77–0.80), and 0.83 (0.82–0.83) at Stanford, Cedars-Sinai, and Columbia respectively. While SEER was trained on the binary 5-year prediction task, it was similarly accurate in predicting relative survival times, achieving Harrell’s C-statistics^20^ of 0.82 (0.80–0.83), 0.78 (0.77–0.78), and 0.81 (0.81–0.081). Using the top tertile as a cutoff, SEER balanced sensitivity and specificity well at 0.76 (0.71–0.81) and 0.75 (0.75–0.76) respectively at Stanford, 0.52 (0.49–0.55) and 0.85 (0.85–0.86) at Cedars-Sinai, and 0.80 (0.80–0.81) and 0.71 (0.70–0.71) at Columbia. It achieved positive predictive values of 0.07 (0.06–0.08), 0.16 (0.15–0.18), and 0.16 (1.15–0.16) at the three sites, i.e., 6–16% of patients in the top third of SEER risk suffered a cardiovascular mortality within 5 years. Being in the top third of SEER indicated a 5.4 (4.6–6.3)-fold age and sex-adjusted increase in hazard of cardiovascular mortality at Stanford. In the Stanford test set, SEER stratified risk across all time scales between a few days and over 10 years (Fig. 1B), and achieved an AUC of 0.80 (0.76–0.84) in predicting 5-year cardiovascular mortality among patients who survived for at least one year (Supplementary Table 4). It also achieved good performance across patients with different underlying rhythms (Supplementary Table 4).

**Table 1:** Performance of SEER in predicting cardiovascular mortality in the three test sets.

		Harrell C-statistic	5-year AUC	Sensitivity	Specificity	Positive Predictive Value	F1 score
Stanford	12-lead	0.815 (0.798–0.826)	0.832 (0.810–0.854)	0.756 (0.709–0.805)	0.753 (0.746–0.760)	0.067 (0.058–0.076)	0.124 (0.108–0.138)
	1-lead	0.781 (0.760–0.793)	0.797 (0.773–0.824)	0.756 (0.710–0.805)	0.672 (0.663–0.680)	0.052 (0.045–0.058)	0.097 (0.085–0.107)
Cedars-Sinai	12-lead	0.777 (0.768–0.783)	0.781 (0.767–0.795)	0.519 (0.487–0.550)	0.851 (0.846–0.856)	0.162 (0.151–0.176)	0.247 (0.232–0.266)
	1-lead	0.771 (0.764–0.778)	0.778 (0.765–0.792)	0.843 (0.822–0.866)	0.567 (0.560–0.573)	0.098 (0.092–0.104)	0.176 (0.166–0.185)
Columbia	12-lead	0.808 (0.806–0.810)	0.825 (0.821–0.829)	0.802 (0.796–0.809)	0.706 (0.704–0.708)	0.156 (0.153–0.159)	0.261 (0.258–0.265)
	1-lead	0.755 (0.753–0.757)	0.761 (0.757–0.765)	0.718 (0.710–0.726)	0.685 (0.683–0.687)	0.132 (0.130–0.135)	0.224 (0.220–0.228)

Patients in the top third of the SEER score were at higher risk for developing a range of incident cardiovascular diseases (Fig. 1C). In the Stanford test set, age and sex-adjusted hazard ratios were 3.2 (2.9–3.5) for incident heart failure, 2.0 (1.7–2.5) for myocardial infarction, 1.6 (1.4–1.9) for stroke, and 4.2 (3.0–5.7) for sudden cardiac death. For a composite of those four events, the adjusted hazard ratio was 2.4 (2.2–2.7). The trend was similar for other cardiovascular conditions including atrial fibrillation (2.2 (2.0–2.5)), heart block (2.8 (2.4–3.2)), cardiomyopathy (4.0 (3.4–4.7)), aortic stenosis (2.3 (1.8–3.0)), and peripheral vascular disease (1.7 (1.5–2.1)). We compared SEER to models trained specifically to predict different future cardiovascular diseases (Supplementary Table 5). SEER performed similarly to models custom-trained to predict 5-year ASCVD and myocardial infarction. In contrast, SEER was outperformed by a heart failure-specific model (0.76 (0.74–0.77) vs 0.82 (0.81–0.83)) and an atrial fibrillation-specific model (0.68 (0.66–0.70) vs 0.752 (0.73–0.77)). All risk scores were strongly correlated with SEER, with Spearman correlations between 0.44 (0.43–0.45; atrial fibrillation) and 0.63 (0.62–0.64; heart failure). All risk scores were strongly correlated with SEER, with Spearman correlations between 0.44 (0.43–0.45; atrial fibrillation) and 0.63 (0.62–0.64; heart failure).

### SEER complements the PCE risk score

We next explored SEER’s performance on the Stanford and Cedars-Sinai PCE comparison sets, comprising outpatients who had no record of a prior cardiovascular disease event at baseline, were non-diabetic, had an LDL cholesterol measurement below 190 mg/dL, and had a blood pressure measurement within the year prior to the ECG. These criteria were selected to closely represent the set of patients eligible for risk screening for long-term cardiovascular disease, and to allow us to compare SEER to the PCE. In these cohorts, SEER predicted cardiovascular mortality with 5-year AUCs of 0.80 (0.76–0.83) and 0.78 (0.72–0.84), and Harrell C-statistics of 0.78 (0.76–0.82) and 0.78 (0.72–0.80) based on a single 12-lead ECG (Table 2). Within the same set, the PCE achieved 5-year AUCs of 0.65 (0.59–0.71) and 0.78 (0.72–0.84) and Harrell C-statistics of 0.66 (0.61–0.67) and 0.76 (0.71–0.80). The PCE’s modest performance as measured by AUC is consistent with what similar studies have found in the past^21^. For predicting low versus moderate risk, SEER achieved a net reclassification improvement (NRI) over PCE of 14.8% among events and 3.0% among non-events, resulting in a total NRI of 17.8%^22^.

**Table 2:** Performance of SEER, the PCE Score, and a composite of the two in the Stanford and Cedars-Sinai comparison sets.

		Cardiovascular Mortality		ASCVD events	
		5-year AUC	Harrell C-statistic	5-year AUC	Harrell C-statistic
Stanford	SEER	0.795 (0.762–0.831)	0.782 (0.759–0.817)	0.668 (0.647–0.687)	0.661 (0.652–0.669)
	PCE	0.651 (0.593–0.707)	0.658 (0.614–0.672)	0.708 (0.689–0.726)	0.703 (0.688–0.714)
	Composite	0.804 (0.771–0.842)	0.795 (0.781–0.817)	0.704 (0.685–0.722)	0.697 (0.687–0.714)
Cedars-Sinai	SEER	0.779 (0.724–0.835)	0.776 (0.724–0.802)	0.632 (0.591–0.674)	0.635 (0.619–0.652)
	PCE	0.787 (0.727–0.859)	0.759 (0.706–0.799)	0.658 (0.615–0.700)	0.642 (0.612–0.668)
	Composite	0.823 (0.769–0.882)	0.811 (0.794–0.861)	0.672 (0.632–0.715)	0.666 (0.643–0.692)

We additionally evaluated SEER’s ability to predict incident hard atherosclerotic cardiovascular disease events (ASCVD) in the PCE comparison sets, using the standard composite endpoint of lethal and non-lethal myocardial infarction, stroke, and sudden cardiac death^3^. SEER achieved a 5-year AUC of 0.67 (0.65–0.69) and Harrell C-statistic of 0.66 (0.65–0.68) in predicting hard ASCVD at Stanford and an AUC of 0.63 (0.59–0.67) and C-statistic of 0.635 (0.62–0.65), while the PCE achieved a 5-year AUC of 0.71 (0.69–0.73) and a better Harrell C-statistic of 0.70 (0.69–0.71) at Stanford and an AUC of 0.66 (0.62–0.70) and a C-statistic of 0.64 (0.61–0.67) at Cedars-Sinai. SEER and the PCE score were only modestly correlated with a Pearson correlation of 0.218 (*P* < 10^−^^195^), and are based on different data modalities.

**Fig. 2:**
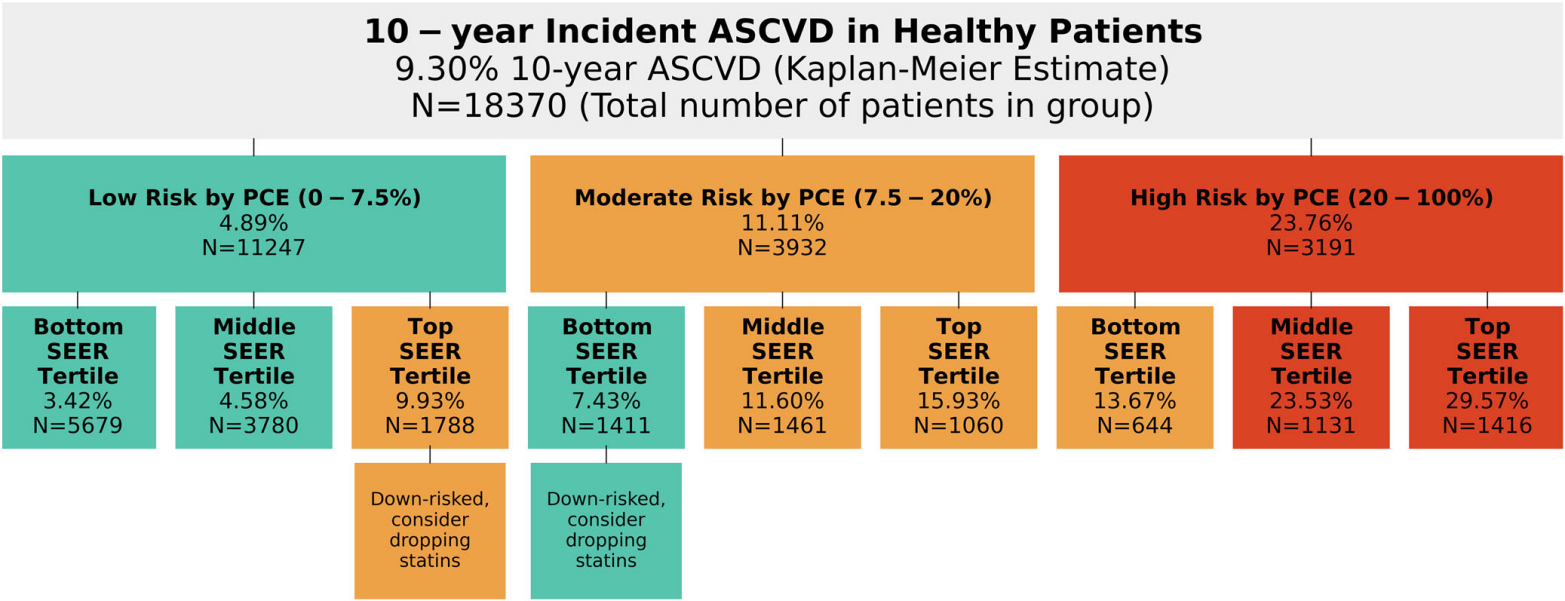
SEER adds value beyond the Pooled Cohort Equations. 10-year incidence of atherosclerotic cardiovascular disease in different groups (Kaplan–Meier estimates) in the Stanford PCE comparison set, with group counts. Top panel is the entire population; middle row of panels are broken down by pooled cohort equation risk; bottom row is broken down by pooled cohort equation risk and SEER risk, dividing at the bottom and top quartiles. Colors represent re-classified binning into low-risk (green), moderate (yellow), and high (red) ASCVD risk according to guidelines.

To understand how SEER might fit into current clinical practice, we next explored how it classified patients versus the PCE score in the Stanford PCE comparison set (Fig. 2). We considered groups determined to be low, moderate, and high risk by the PCE risk score, and examined how SEER would have classified them. We used SEER to separate patients into three tertiles of risk based on cutoffs at the bottom and top thirds of the cross-validation set. The 11,247 patients categorized as low-risk by the PCE risk score (with a PCE-estimated 10-year ASCVD rate below 7.5%^3^) had an actual 10-year ASCVD rate of 4.89% (Kaplan–Meier estimate; 95% CI 4.31%–5.55%) and a 10-year cardiovascular mortality rate of 1.04% (0.79%–1.38%). Within that group, the 1788 patients with a SEER score in the top tertile had a 10-year ASCVD rate of 9.93% (8.03%–12.24%), above the 7.5% cutoff for recommending statins, and a significantly higher cardiovascular mortality rate of 3.54% (2.45%–5.11%). SEER therefore reclassified 16% of patients classified as low risk by PCE into a moderate risk category, identifying additional patients who may benefit from statin therapy. SEER is also able to reclassify patients with moderate 10-year ASCVD risk. Among the 3932 patients with moderate ASCVD risk (7.5–20%) according to the PCE score, the 1411 with a low SEER risk score had a 10-year ASCVD rate of 7.43% (5.73%–9.60%), while the 1060 with high SEER risk had a rate of 15.93% (12.51%–20.17%). Over 35% of patients were reclassified as being slightly below the 7.5% statin cutoff, and over 25% had a higher risk than the moderate PCE risk group overall. Those patients were similarly stratified with respect to cardiovascular mortality risk. SEER also stratified patients with high PCE scores, with patients with low SEER risk experiencing a 13.67% (10.03%–18.48%) 10-year ASCVD rate versus 29.57% (25.34%–34.32%) for patients with high SEER risk. In the Stanford test set the up-risking and stratification trends were generally similar, but the number of patients in each subgroup was much smaller (Supplementary Fig. 3). Risk of cardiovascular mortality followed a similar pattern of up and down-risking (Supplementary Fig. 4). When combined with a weighted average, the composite score achieved an AUC of 0.80 (0.77–0.84) at Stanford and 0.82 (0.77–0.88) at Cedars-Sinai in predicting cardiovascular mortality (Table 2). The optimal weighted average was found to be 16 parts unnormalized SEER score to 1 part unnormalized PCE.

### SEER performs well across diverse populations

**Fig. 3:**
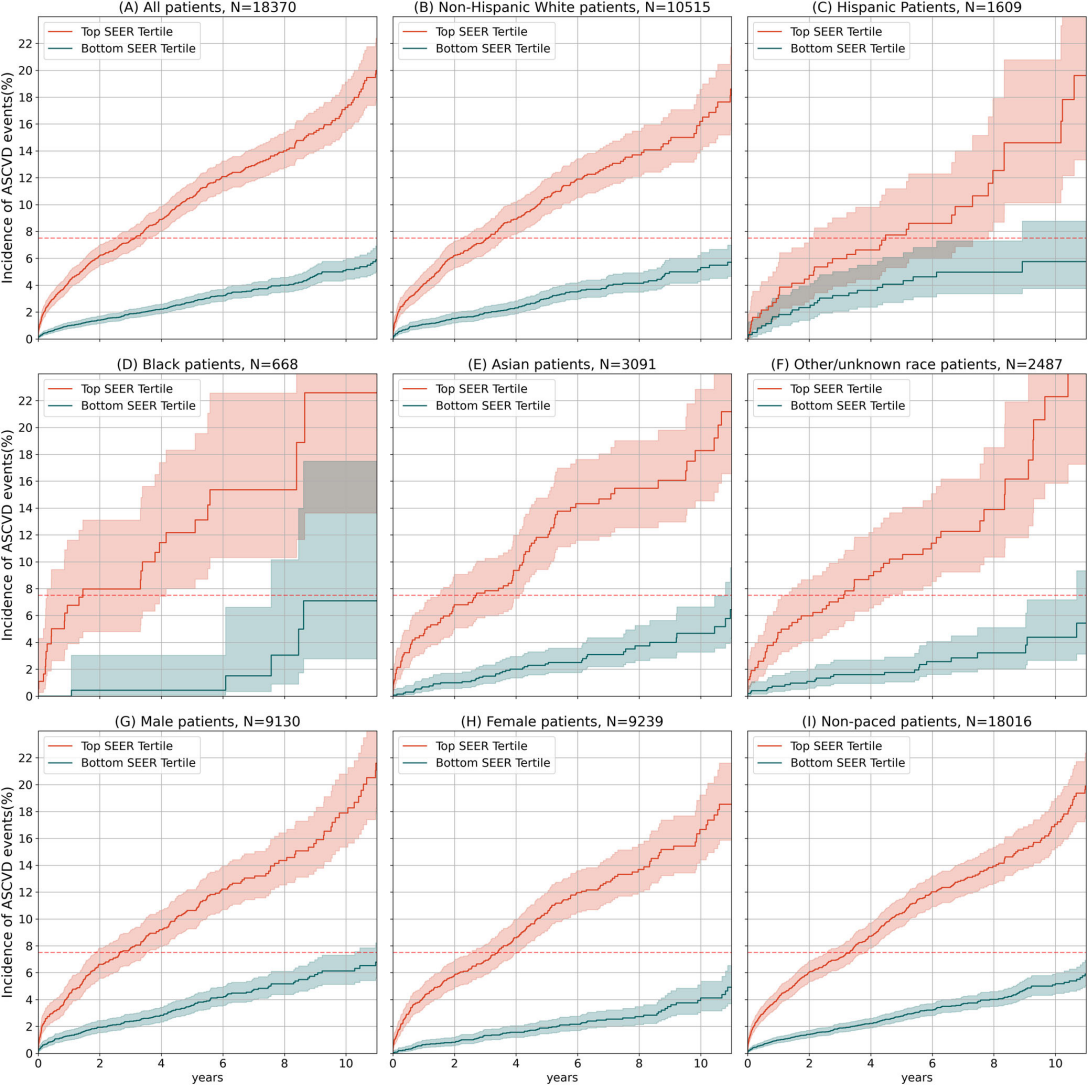
SEER stratifies patients across time and subgroups. **A**–**I** Cumulative incidence of atherosclerotic cardiovascular disease in the Stanford PCE comparison set (Kaplan–Meier estimates), among patients in various demographic groups. The blue and red lines represent the bottom and top third of patients as ranked by SEER. The dotted red line shows the 7.5% risk cutoff used in the decision to prescribe statins.

To understand potential biases in SEER, we performed additional validation on a range of demographic subgroups in the Stanford test set (Supplementary Table 4). To mirror expected clinical use, we set a cutoff for positive prediction at the top third of patients and compared sensitivity and specificity. For all race, ethnicity and sex sub-groups, performance was not significantly different from the entire population. We additionally compared based on AUC and found no significant difference between groups. Survival curves for ASCVD for various demographic groups are shown in Fig. 3, demonstrating robust differentiation across groups.

### SEER-based risk correlates with high-risk ECG and clinical features

**Fig. 4:**
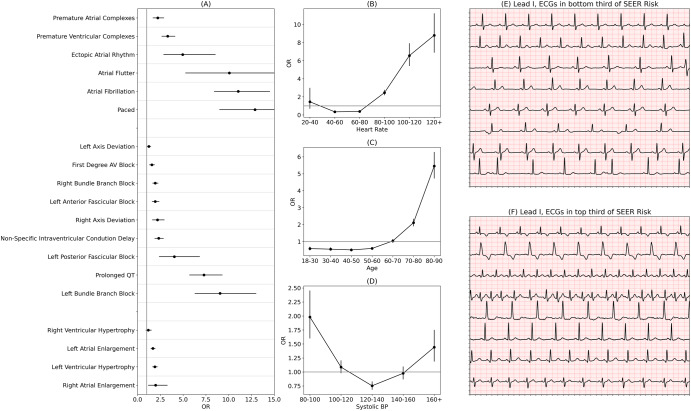
SEER is consistent with known ECG risk factors. **A** Age and sex-corrected odds ratios for being in the top third of SEER risk, given each diagnosis present in the electrocardiogram cardiologist overread, in the Stanford test set. **B**–**D** Age and sex-corrected odds ratios for falling in each bin of three continuous variables (age is not age-corrected). **E** Examples from 8 different patients from the bottom third of SEER risk, lead I. **F** Examples from 8 different patients from the top third of SEER risk, lead I. All error bars represent 95% bootstrap confidence intervals. All error bars represent 95% bootstrap confidence intervals.

Understanding how well-known ECG risk markers affect the SEER score is a challenge, as the model does not take them directly as inputs (for example, the model does not receive a binary “atrial fibrillation” label, but rather a waveform from which it might extract features related to heart rate variance). To address this issue, we utilized odds ratios to interpret the outputs of the model. For each of 16 features parsed from the ECG physician overread, we calculated the age and sex-adjusted odds ratio of falling in the top third of SEER scores given each clinician overread-based diagnosis in the Stanford test set (Fig. 4A). All odds ratios were above 1. The features with lowest odds ratios were right ventricular hypertrophy, left axis deviation, and first-degree AV block with odds ratios of 1.19 (0.93–1.50), 1.24 (1.08–1.43), and 1.56 (1.32–1.86). The highest were atrial flutter, atrial fibrillation, and pacing, with odds ratios of 10.1 (5.27–19.2), 11.1 (8.43–14.5), and 12.9 (9.01–18.4). To understand the degree to which SEER relies on standard ECG markers, we trained a random forest model on 38 standard ECG markers (listed in Supplementary Table 6), which achieved an AUC of 0.68 (0.65–0.70) in predicting 5-year cardiovascular mortality (Supplementary Table 7; versus SEER’s AUC of 0.82).

Increased and severely decreased heart rate both were associated with higher SEER risk, (Fig. 4B), reflecting the elevated risk associated with bradycardia and tachycardia. SEER also correlated with some continuous PCE risk factors. It closely reflected the true risk of elevated age (Fig. 4C). Still, it was not completely driven by age, with a Pearson correlation of 0.18 (*P* < 10^−^^139^). Increased risk among 20–40 year olds was likely due to ascertainment bias of only sicker young patients receiving ECGs. SEER also captured the risk of low blood pressure (Fig. 4D) associated with heart failure, but only predicted a slight increase in risk for elevated blood pressure.

In Fig. 4E, F we show 8 randomly selected low-risk and high-risk ECG examples. Qualitatively, the ECGs in Fig. 4E appear mostly normal: all ECGs are sinus rhythm, and the only abnormalities noted are premature complexes and 1st degree AV Block. Conversely, the ECGs in Fig. 4F show a range of arrhythmias and morphologic abnormalities.

### SEER performs well using a single ECG lead and outperforms more limited data

The main SEER model makes predictions based on 12-lead ECG waveforms. To understand which features are important for prediction, we additionally trained and evaluated models on more limited input data. Using only lead I of each ECG, SEER was still able to predict 5-year cardiovascular mortality in all patients with an AUCs of 0.78 (0.76–0.79), 0.77 (0.76–0.78), and 0.76 (0.75–0.76) in the three test sets, at most a 0.06 drop in AUC from the 12-lead model (Table 1). Supplementary Fig. 5 reproduces Fig. 1 using the single-lead model. A random forest model based on the 11 common ECG parameters (Supplementary Table 6) generated by the Philips Tracemaster software achieved an AUC of 0.71 (0.66–0.75) and a Harrell’s C-statistic of 0.70 (0.67–0.73; Supplementary Table 7) in the Stanford test set. This result suggests that SEER makes substantial use of features other than the ones used in standard ECG algorithms.

## Discussion

In this work we have presented SEER, the Stanford Estimator of ECG Risk, a deep neural network for estimating long-term risk of cardiovascular disease and mortality from the 12-lead ECG. We evaluated its performance in three held-out test sets and two PCE comparison sets from three different institutions with two different ECG vendors, demonstrating robust accuracy across sites and vendors. We demonstrated its performance relative to the existing PCE risk score, the most commonly used method in the United States for estimating long-term cardiovascular risk and guiding lipid therapy. We showed that SEER encompasses known ECG risk factors, but also performs at a level beyond a model based solely on those risk factors. SEER also performed well when trained and evaluated on single-lead ECGs.

We have demonstrated our risk score’s utility in improving the stratification of 10-year ASCVD risk through use alongside the PCE. SEER uncovers 16% of patients misclassified as low-risk by the PCE, highlighting a new cohort of patients who may benefit from statins and would be missed following current practices. SEER also stratifies patients with intermediate risk according to the PCE score, showing potential to perform a similar role to what the CAC score currently plays^23^. Given the advantages of ECGs over CAC scans—lower expense, lack of radiation, clinical ubiquity, and presence in resource-limited environments – SEER or similar models could potentially present an attractive alternative after further validation. Our model is similar in accuracy to that of the CAC score^23,24^, which in one study achieved an AUC of 0.702 in predicting 5-year, all-cause mortality^25^. Given the strong performance based on a single-lead hospital ECG, it is also likely that SEER could also be deployed on smartwatch ECGs for widespread screening. And the model’s ability to predict a range of cardiovascular disease, including heart failure and cardiomyopathy, make it potentially more broadly applicable past screening for ASCVD and cardiovascular mortality risk.

Previous studies^6,26^ have estimated the benefit of different strategies for improving targeted statin usage, in terms of estimated reductions in mortalities. Closely following their methods, we estimate that following a conservative up-risk-only strategy, if SEER up-risked 10.1% (9.6–10.7%) of the population 40-75 without diabetes from statin-ineligible to statin-eligible, it would make eligible 20,405 (19,331–21,479) patients per year who are at risk of cardiovascular death. Given 100% statin uptake, this could prevent an additional 3468 (775–6029) cardiovascular deaths per year in the US. With a more conservative 63% uptake, 2185 (487–3789) cardiovascular mortalities would be averted.

We selected 5-year cardiovascular mortality as the training outcome to study for several reasons. Cardiovascular mortality is ultimately the main outcome that cardiovascular screening and interventions such as statin therapy aim to prevent. We chose a 5-year cutoff to balance our goal of predicting longer-term events with the availability of followup data, given that only a fraction of the ECGs in our dataset were taken more than ten years ago. We focused our comparison to the PCE score on 10-year ASCVD prediction (making use of Kaplan-Meier estimates) to contextualize our model within current clinical guidelines.

This study has a few key limitations, which also present directions for future work. While we tested SEER retrospectively across three different hospital systems, prospective trials are necessary to verify how the score will perform in clinical practice. Large cohort studies could provide a good evaluation group, but most either do not have available digitized ECGs or do not have sufficient followup to allow for evaluation. SEER was trained on data from only one medical center, and thus may suffer from demographic biases based on the specific training population, although we did not find major differences in performance across demographic groups. And while we presented results on single-lead hospital ECGs, additional evaluation is required to fully understand how SEER would perform on smartwatch data. Finally, while we had sufficient followup data to compute Kaplan-Meier estimates of ten-year survival, there was insufficient data to compute AUC and other metrics at a 10-year cutoff.

Our risk score, SEER, identifies groups of patients to be up-risked and successfully stratifies intermediate risk patients for risk of ASCVD and cardiovascular mortality. Given that cardiovascular disease is a leading cause of death in the United States and globally, if fully deployed alongside the PCE score and other risk tools SEER could lead to a substantial decrease in mortality through more accurate risk stratification. The relative low-cost and ubiquity of the ECG makes common application possible.

## Methods

### Study populations and data sources

SEER was trained, developed, and evaluated using a dataset of resting ECGs from Stanford University Medical Center (Stanford) consisting of all non-low quality ECGs from patients above the age of 18 taken during the course of clinical care between March 2008 and May 2018. In total we extracted 910,966 ECGs from 307,557 patients from the Phillips TraceMaster system (Supplementary Table 1, Supplementary Fig. 1). All ECGs were saved as 10 s signals from all 12 leads of the ECG, sampled at 500 Hz. We applied band pass and wandering baseline filters to the signals, normalized on a per-lead basis, and downsampled to 250 Hz for performance reasons and to match data from other sites. Measurements and text overreads were also extracted from TraceMaster, and ECG diagnoses were extracted from text cardiologist overreads using string matching. ECGs were randomly partitioned by patient into the training/cross-validation, validation, and test sets in an 8:1:1 ratio. For training and validation, we only considered ECGs with either a cardiovascular mortality (defined below) within 5 years after the ECG, or more than 5 years of followup after the ECG (defined in detail below), resulting in 311,334 (38,970) ECGs in the training (validation) set. Model parameters were fit using the training set, and hyperparameters were chosen based on the validation set. All ECGs from each patient were used during model training. All model development, training, and hyperparameter selection was performed using this split. The Stanford University Institutional Review Board approved this study under protocol 41,045 and it complied with all relevant ethical regulations; the review board waived the requirement for informed consent owing to the retrospective nature of the data and project.

During model evaluation, we only considered the first ECG from each patient. Once a final model was selected, we performed 8-fold cross-validation on the training set to obtain model predictions on 244,839 ECGs from a set of 244,839 patients who were not part of the validation or test set (not all of whom had 5 years of followup), which we refer to as the cross-validation set. Cross-validation predictions on each fold were generated based on models trained on all other cross-validation folds and the validation set. We additionally report results on the PCE comparison set, the subset of the cross-validation set consisting of 18,370 non-inpatients who had no record of a prior cardiovascular disease event at baseline, were non-diabetic, and had an LDL cholesterol measurement below 190 mg/dL and any blood pressure measurement within the year prior to the ECG.

To understand how SEER performs on a range of populations, we additionally evaluated SEER on three held out test sets from Stanford, Cedars-Sinai Medical Center (Cedars-Sinai), and Columbia University Irving Medical Center (Columbia; Supplementary Fig. 2). The Stanford test set consists of 31,899 first resting ECGs, from patients not in the Stanford training or validation sets. The Cedars-Sinai test set consists of 46,095 first resting ECGs taken at Cedars-Sinai from the General Electric MUSE system, with mortality and event data from EPIC Clarity. The Columbia test set consists of 458,455 ECGs first resting ECGs taken at Columbia from the General Electric MUSE system, with mortality and event data from their OMOP database. Demographic data for the Cedars-Sinai and Columbia test sets are in Supplementary Tables 2 and 3. We additionally created the Cedars-Sinai PCE comparison set, with same inclusion criteria as the Stanford PCE comparison set but derived as a subset of the Cedars-Sinai test set, with 4065 ECGs.

Followup mortality and disease data were queried from STARR-OMOP^27^, a common data model for accessing Stanford electronic health records, and extended to December of 2020 for model training and February of 2022 for evaluation. During evaluation we supplemented mortality data from Stanford’s health record system with data from the social security death index. Our primary outcome of interest was cardiovascular mortality, defined following previous work^28^ as a mortality in the EHR falling within thirty days of a condition-record of myocardial infarction, ischemic stroke, intracranial hemorrhage, sudden cardiac death, or hospitalization for heart failure. During training we only considered ECGs with a cardiovascular mortality within 5 years of the ECG or 5 years of followup after the ECG, defined as a measurement, admission, or mortality more than 5 years after the ECG. The same definition was used for censoring times in survival analyses, but the 5-year cutoff was not applied, so as to understand how SEER performs in both shorter and longer timespans. The same OMOP queries were used on Columbia’s OMOP database to pull outcomes. Separate queries were written for Cedars-Sinai’s EPIC Clarity-based system.

Additional data was queried from STARR-OMOP as selection criteria and for the computation of the PCE risk score. Blood pressure and cholesterol measurements were taken within the year prior to the ECG. Smoking, diabetes, and antihypertensive status were determined using any label prior to the ECG, and in the case where there was no prior label were by default set to false. Atherosclerotic cardiovascular disease was defined as the first incidence of myocardial infarction, ischemic stroke, intracranial hemorrhage, or sudden cardiac death in the electronic health record. Atrial fibrillation, heart block, cardiomyopathy, pulmonary artery disease, and aortic stenosis were all defined as the first incidence in the electronic health record. OMOP concept codes for all conditions and measurements are shown in Supplementary Table 8. The PCE are a proportional hazards model, meaning that they order patients by risk in the same order across different time scales. Thus, computing 5-year AUROC and any other ordering-based metric does not require adjustment. Similarly and using the same proportional hazards assumptions, training a model to predict 5-year cardiovascular mortality should also correctly order patients by ten-year cardiovascular mortality risk.

### Model development and training

We trained a convolutional neural net to predict 5-year cardiovascular mortality among ECGs with either a positive event within 5 years or a record in the EHR more than 5 years afterwards. Model development was performed using Python 3.9 and PyTorch 1.11, and models were trained on single Nvidia Titan Xp GPUs using Stanford’s Sherlock computing cluster. We explored several convolutional architectures and chose the one yielding the highest validation accuracy, described in Supplementary Fig. 6 and similar to architectures described in previous work^15^. Convolutional architectures are well-suited to ECG data due to the repetition of motifs across time and examples, allowing for convolutional filters to be fit to share information and reduce complexity. The model was chosen and all hyperparameters were tuned by training on the training set and evaluating on the validation set. We used a batch size of 128, a weight decay hyperparameter of 10^−^^4^, and the ADAM optimizer^29^. We initialized the learning rate to 10^−^^3^ and reduced it by a factor of 10 each time the validation loss plateaued for more than five epochs, and stopped training once the learning rate fell to 10^−^^6^. Models were selected based on area under the receiver operator characteristic curve (AUC). We explored a number of model architectures from previous papers and a range of hyperparameters and selected the ones best-performing on the validation set.

Once a model and hyperparameters were chosen, we trained eight more models using cross-validation on the training and validation sets to generate model predictions on the portions of the training set not used to evaluate the model during training. We averaged the results of those eight models and the original model to make predictions on the test set. All results are based on models trained at Stanford. ECGs from Cedars-Sinai Medical Center and Columbia Medical Center were treated exactly as ECGs at Stanford, downsampled from 500 to 250 Hz, pre-processed using band pass and high pass wandering baseline filters, and normalized per-lead, based on normalization parameters specific to Cedars-Sinai. Both Cedars-Sinai and Columbia use the General Electric MUSE ECG software and General Electric ECG machines.

We converted the continuous model prediction to a categorical risk prediction by taking the two tertiles of the SEER score in the Stanford cross-validation set (i.e., the 33.3… and 66.6… percentiles). All references to bottom and top thirds of model predictions are based on the cutoffs from this group, including validation at other sites and experiments in the Stanford PCE comparison set. These cutoffs are equivalent to 1.1% and 3.9% risk of cardiovascular mortality (which should not be directly compared to 10-year risk of ASCVD).

Single lead ECG models were trained using the same architecture and hyperparameters as 12-lead models, but using only lead I of the ECG and using 1 by 1 convolutions in place of the 1 by 12 convolutions. Random forest models were developed and trained using XGBoost 1.5, using the features in Supplementary Table 6.

### Statistical analysis

We primarily compared models based on the area under the receiver operator characteristic (AUC) and the Harrell’s C-statistic^20^. The former is a standard metric used for evaluating stratification in binary classification tasks, while the latter is a similar score for evaluating stratification in survival prediction tasks with censoring. The AUC was computed using the scikit-learn Python package, and 95% confidence intervals were constructed using the bootstrap method with 100 samples. Unless otherwise noted, all binary metrics were computed at a 5-year time horizon, comparing all examples with an event within 5 years versus all examples with no event but other followup data after 5 years. The c-statistic was computed using the lifelines Python package, and 95% confidence intervals were constructed using the bootstrap method with 100 samples. C-statistics were computed including the entire population. We additionally compute sensitivity, specificity, and positive predictive values using standard definitions and using the top tertile as the cutoff for positive predictions, and NRI based on up-risking the top tertile and downrisking the bottom tertile.

We computed hazard ratios to measure how predictive SEER is of future outcomes, and odds ratios to measure how current ECG and clinical features affect SEER. Hazard ratios were calculated using the lifelines Python package using Cox proportional hazards models, correcting for age and sex. All hazard ratios indicate the hazard of a patient being in the top third of SEER risk. Kaplan–Meier estimates were computed using the lifelines Python package. All confidence intervals on Kaplan-Meier curves are 95% Kaplan–Meier confidence intervals. The observed event rates in Fig. 2 are Kaplan–Meier estimates, since the high number of censoring events would otherwise bias the event rates to be higher. Odds ratios were calculated using the statsmodels Python package using logistic regression models correcting for age and sex (with the exception of the odds ratios for age, which were only corrected for sex). All odds ratios indicate the odds of a patient being in the top third of SEER risk given the characteristic.

### Reporting summary

Further information on research design is available in the Nature Research Reporting Summary linked to this article.

### Supplementary information

Reporting Summary

Supplementary Material

## Data Availability

The raw patient data is not publicly available due to institutional policy and human subjects approval to protect patient privacy.

## Code availability

Code and model weights for training and running SEER are available at github.com/weston100/AI-ECG.
