# Supplementary material for: A deep learning-based electrocardiogram risk score for long term cardiovascular death and disease
Source: NPJ Digit Med. 2023 Sep 12;6:169. doi: 10.1038/s41746-023-00916-6 (PMC10497604; doi:10.1038/s41746-023-00916-6)
Supplement: Supplementary file 2 — Supplementary Material [file 41746_2023_916_MOESM2_ESM.pdf]

Supplementary Figures

Supplementary Figure 1. Consort diagram of Stanford population.

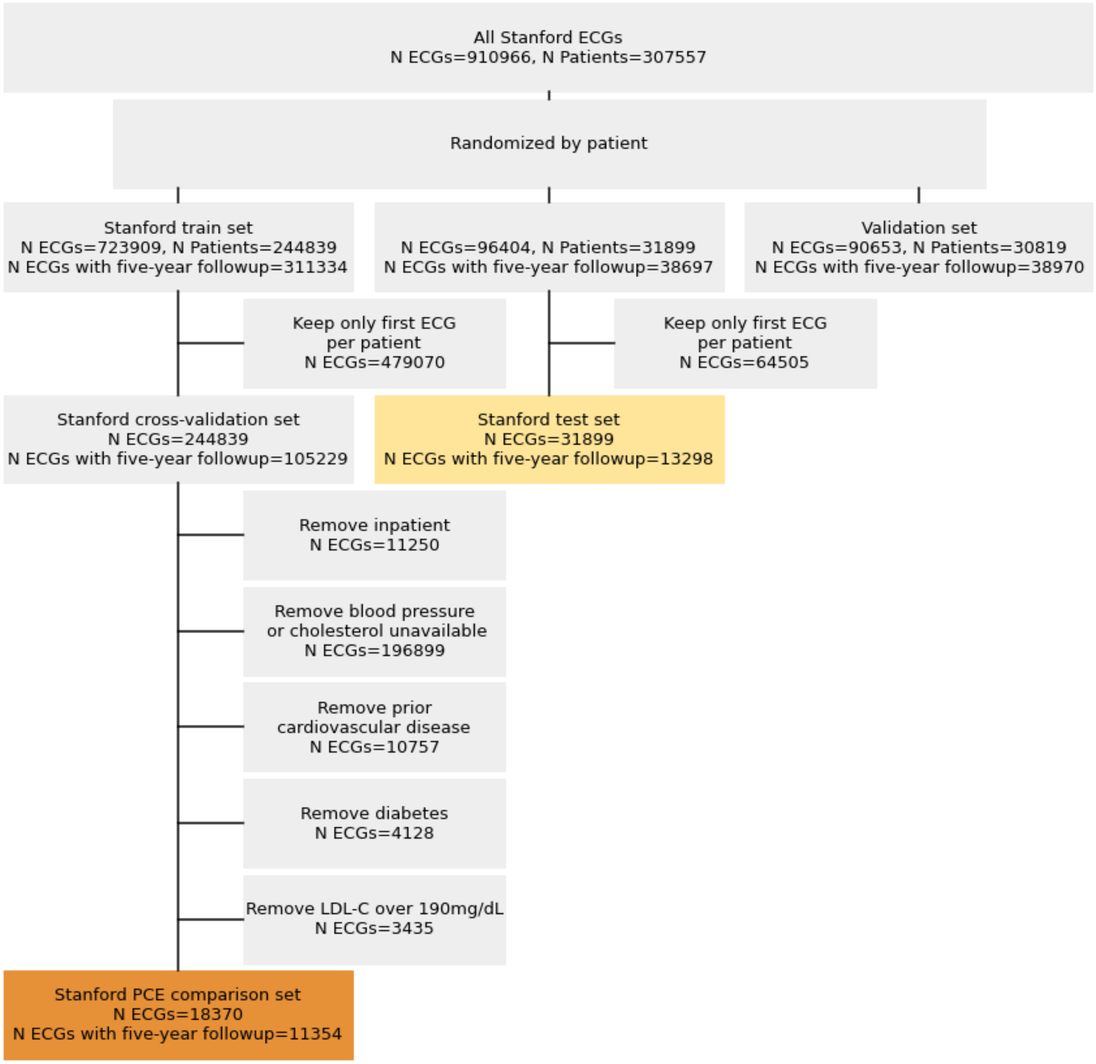

Supplementary Figure 2. Consort diagrams of Cedars-Sinai and Columbia populations.

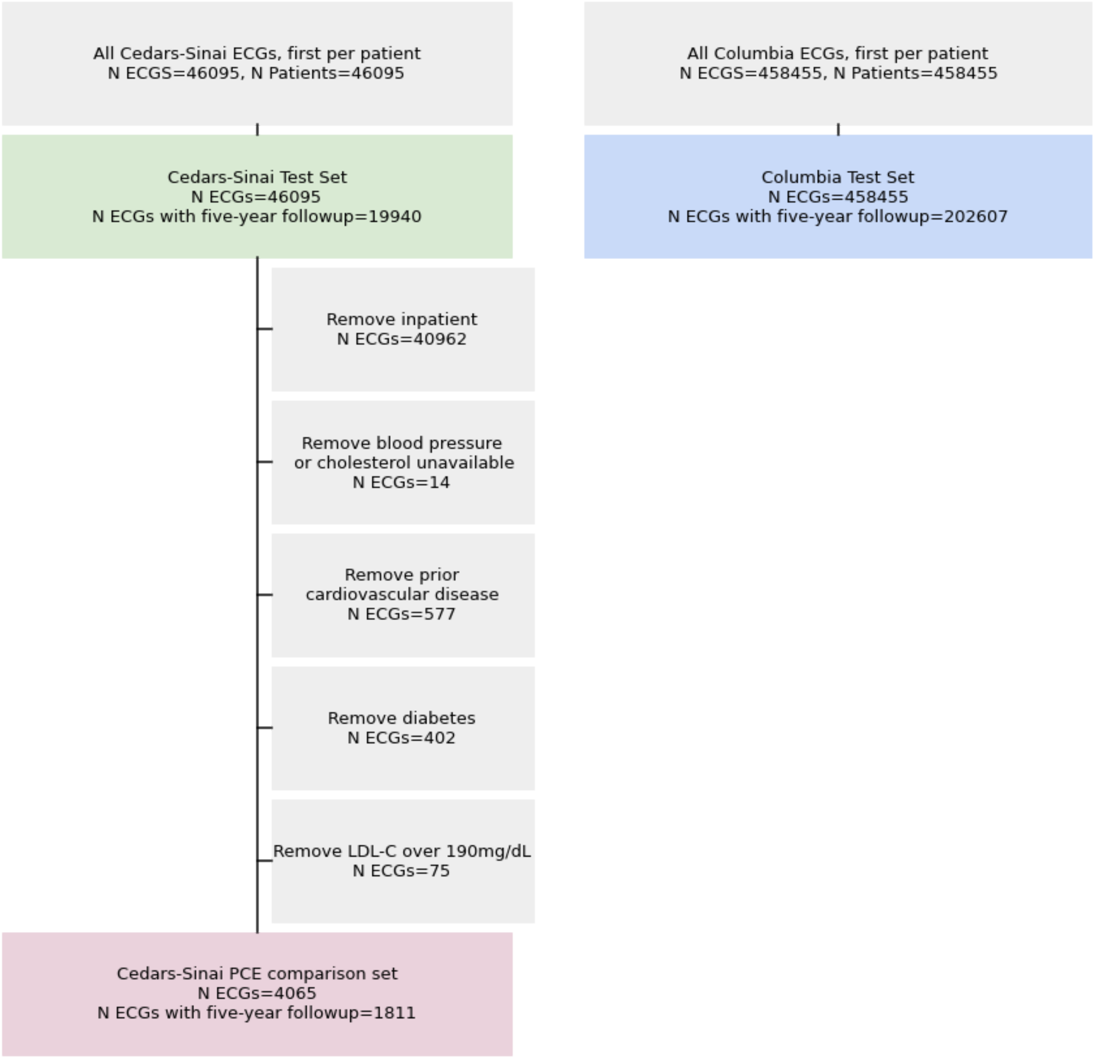

Supplementary Figure 3. SEER adds value beyond the Pooled Cohort Equations in The Stanford Test Set. 10-year incidence of atherosclerotic cardiovascular disease in different groups (Kaplan-Meier estimates) in the Stanford test set applying the same inclusion criteria as the PCE comparison sets, with group counts. Top panel is the entire population; middle row of panels are broken down by pooled cohort equation risk; bottom row is broken down by pooled cohort equation risk and SEER risk, dividing at the top and bottom quartiles. Colors represent re-classified binning into low-risk (green), moderate (yellow), and high (red) ASCVD risk according to guidelines.

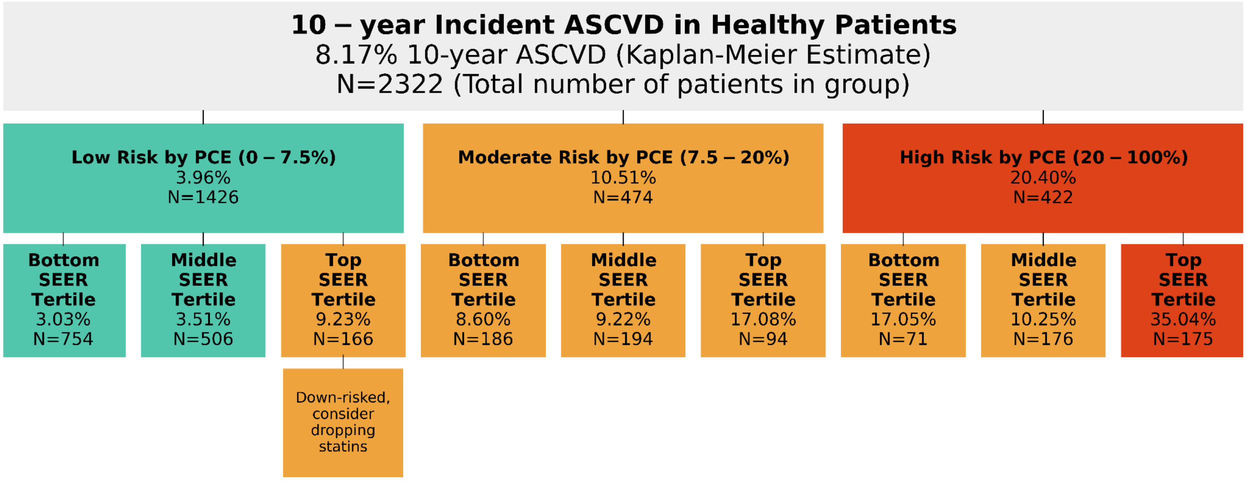

Supplementary Figure 4. SEER adds value beyond the Pooled Cohort Equations In Predicting Mortality. 10-year incidence of cardiovascular mortality in different groups (Kaplan-Meier estimates) in the Stanford PCE comparison set, with group counts. Top panel is the entire population; middle row of panels are broken down by pooled cohort equation risk; bottom row is broken down by pooled cohort equation risk and SEER risk, dividing at the top and bottom quartiles. Colors represent re-classified binning into low-risk (green), moderate (yellow), and high (red) ASCVD risk according to guidelines.

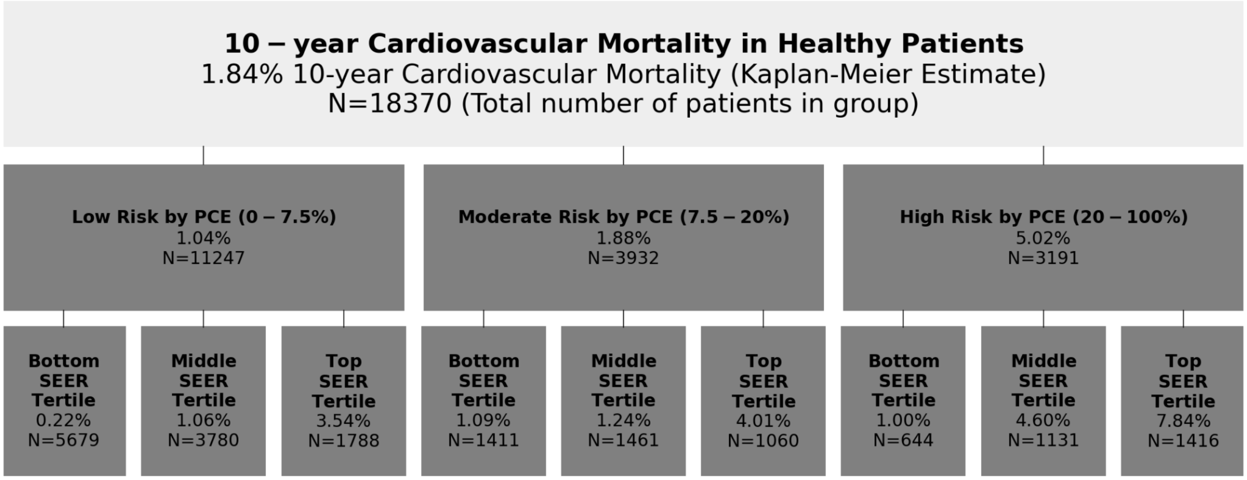

Supplementary Figure 5. Diagram of the SEER Model.

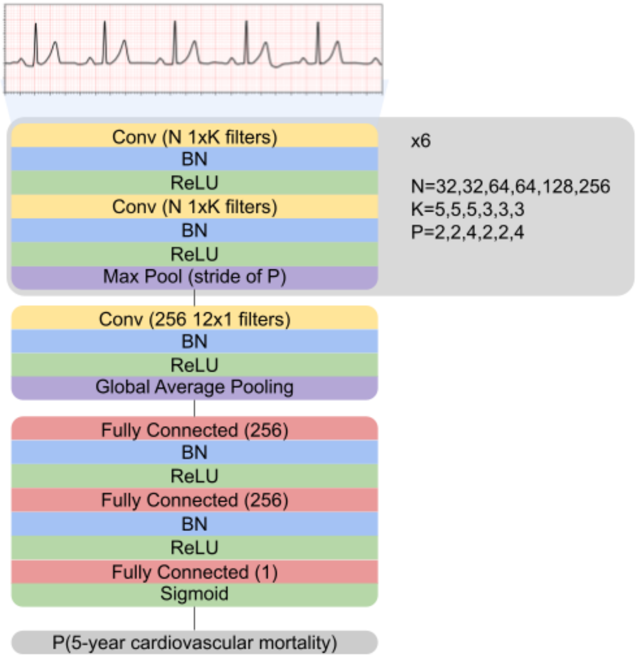

Supplementary Figure 6. Performance of the single-lead SEER Model. Same as figure 1, but based on single lead model. (A) Receiver Operator Characteristic (ROC) curves and Areas Under the Curve (AUCs) for Stanford, Cedars-Sinai, and Columbia test sets. (B) Cumulative incidence of cardiovascular mortality in the Stanford PCE comparison set (Kaplan-Meier estimates). The blue and red lines represent the bottom and top third of patients as ranked by SEER; the black line represents all patients. (C) Hazard ratios of various cardiovascular diseases given that a patient is in the top tertile of SEER risk, in the Stanford test set. (D-F) Cumulative incidence of atherosclerotic cardiovascular disease in the Stanford PCE set (Kaplan-Meier estimates), among patients called low-risk (0-7.5%) moderate risk (7.5%-20%), and high-risk (20-100%) by the PCE. The blue and red lines represent the bottom and top tertiles of patients as ranked by SEER; the black line represents all patients. The dotted red line shows the 7.5% risk cutoff used in the decision to prescribe statins. All error bars represent 95% bootstrap confidence intervals.

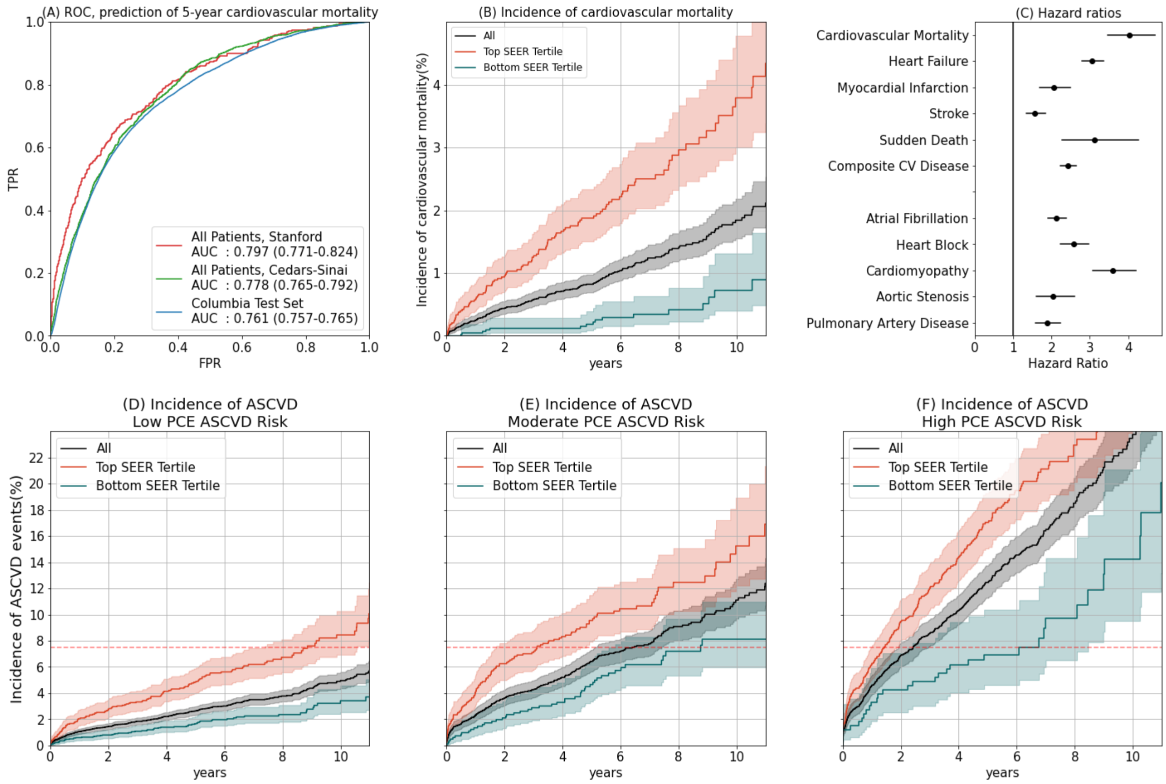

Supplemental Tables

Supplementary Table 1. Demographics in Stanford University Medical Center test set.

	Full dataset	Model Training Set	Model Validation Set	Model Training and Validation Set	Cross-Validation set	PCE comparison set	PCE comparison set, Bottom SEER Tertile	PCE comparison set, Middle SEER Tertile	PCE comparison set, Top SEER Tertile	Test set
Number of ECGs	910966	723909	90653	814562	244839	18370	7734	6372	4264	31899
Number of patients	307557	244839	30819	275658	244839	18370	7734	6372	4264	31899
Number of ECGs with five-year follow up	389001	311334	38970	350304	105229	11354	4857	3981	2516	13298
Five-year cardiovascular mortality	28680 (3.15%)	23177 (3.20%)	2778 (3.06%)	25955 (3.19%)	2435 (0.99%)	128 (0.70%)	9 (0.12%)	35 (0.55%)	84 (1.97%)	307 (0.96%)
Ten-year cardiovascular	33015 (3.62%)	26705 (3.69%)	3264 (3.60%)	29969 (3.68%)	2957 (1.21%)	189 (1.03%)	17 (0.22%)	55 (0.86%)	117 (2.74%)	357 (1.12%)

mortality										
Prior ASCVD	119908 (13.16%)	95431 (13.18%)	11629 (12.83%)	107060 (13.14%)	12266 (5.01%)	0 (0.00%)	0 (0.00%)	0 (0.00%)	0 (0.00%)	1563 (4.90%)
Five-year ASCVD	37540 (4.12%)	29811 (4.12%)	3781 (4.17%)	33592 (4.12%)	6650 (2.72%)	819 (4.46%)	181 (2.34%)	256 (4.02%)	382 (8.96%)	836 (2.62%)
Ten-year ASCVD	47033 (5.16%)	37347 (5.16%)	4887 (5.39%)	42234 (5.18%)	8656 (3.54%)	1059 (5.76%)	241 (3.12%)	348 (5.46%)	470 (11.02%)	1074 (3.37%)
Age	60.45 (17.87)	60.43 (17.87)	60.53 (17.82)	60.44 (17.86)	56.44 (18.49)	55.85 (17.04)	51.43 (15.39)	56.53 (16.78)	62.85 (17.75)	56.43 (18.58)
Below 40	113489 (12.46%)	90174 (12.46%)	11201 (12.36%)	101375 (12.45%)	44247 (18.07%)	3537 (19.25%)	1830 (23.66%)	1153 (18.09%)	554 (12.99%)	5794 (18.16%)
Above 75	177394 (19.47%)	140798 (19.45%)	17614 (19.43%)	158412 (19.45%)	36744 (15.01%)	2519 (13.71%)	425 (5.50%)	875 (13.73%)	1219 (28.59%)	4813 (15.09%)
Female	363450.0 (47.05%)	288187.0 (46.92%)	36819.0 (47.73%)	325006.0 (47.01%)	108906.0 (50.12%)	9239.0 (50.29%)	3634.0 (46.99%)	3420.0 (53.67%)	2185.0 (51.24%)	14263.0 (50.42%)
Non-Hispanic White	416714 (45.74%)	330783 (45.69%)	41994 (46.32%)	372777 (45.76%)	111728 (45.63%)	10515 (57.24%)	4309 (55.72%)	3700 (58.07%)	2506 (58.77%)	14422 (45.21%)
Hispanic White	94753 (10.40%)	75564 (10.44%)	9238 (10.19%)	84802 (10.41%)	26290 (10.74%)	1609 (8.76%)	642 (8.30%)	572 (8.98%)	395 (9.26%)	3515 (11.02%)
Asian	106296.0 (13.76%)	84266.0 (13.72%)	10304.0 (13.36%)	94570.0 (13.68%)	29189.0 (13.43%)	3091.0 (16.83%)	1391.0 (17.99%)	1023.0 (16.05%)	677.0 (15.88%)	3874.0 (13.69%)
Black	3399	26857.0	3781.0	30638.0	8874.0	668.0	250.0	232.0	186.0	1130.

	7.0 (4.40%)	(4.37%)	(4.90%)	(4.43%)	(4.08%)	(3.64%)	(3.23%)	(3.64%)	(4.36%)	0 (3.99%)
Other/unknown race	199073 (21.85%)	159532 (22.04%)	19214 (21.20%)	178746 (21.94%)	62551 (25.55%)	3732 (20.32%)	1614 (20.87%)	1288 (20.21%)	830 (19.47%)	8198 (25.70%)
Smoking	6766.0 (0.74%)	5363.0 (0.74%)	661.0 (0.73%)	6024.0 (0.74%)	1867.0 (0.76%)	198.0 (1.08%)	113.0 (1.46%)	50.0 (0.78%)	35.0 (0.82%)	244.0 (0.76%)
Smoking missing	315534 (34.64%)	250812 (34.65%)	31378 (34.61%)	282190 (34.64%)	101119 (41.30%)	766 (4.17%)	351 (4.54%)	243 (3.81%)	172 (4.03%)	13052 (40.92%)
Hispanic patients	96987.0 (12.56%)	77384.0 (12.60%)	9402.0 (12.19%)	86786.0 (12.55%)	26950.0 (12.40%)	1658.0 (9.03%)	663.0 (8.57%)	588.0 (9.23%)	407.0 (9.55%)	3603.0 (12.74%)
Prior HF	143190 (15.72%)	113114 (15.63%)	14630 (16.14%)	127744 (15.68%)	10779 (4.40%)	0 (0.00%)	0 (0.00%)	0 (0.00%)	0 (0.00%)	1393 (4.37%)
Prior MI	77348 (8.49%)	61582 (8.51%)	7288 (8.04%)	68870 (8.45%)	6467 (2.64%)	0 (0.00%)	0 (0.00%)	0 (0.00%)	0 (0.00%)	856 (2.68%)
Prior Stroke	46979 (5.16%)	37605 (5.19%)	4815 (5.31%)	42420 (5.21%)	5788 (2.36%)	0 (0.00%)	0 (0.00%)	0 (0.00%)	0 (0.00%)	716 (2.24%)
Prior CV disease	206114 (22.63%)	163250 (22.55%)	20728 (22.87%)	183978 (22.59%)	20449 (8.35%)	0 (0.00%)	0 (0.00%)	0 (0.00%)	0 (0.00%)	2620 (8.21%)
Diabetes	130911 (14.37%)	104539 (14.44%)	12546 (13.84%)	117085 (14.37%)	18338 (7.49%)	0 (0.00%)	0 (0.00%)	0 (0.00%)	0 (0.00%)	2437 (7.64%)
Hypertensio	300896	238835 (32.99%)	29974 (33.06%)	268809 (33.00%)	83706 (34.19	9453 (51.46%)	3792 (49.03%)	3424 (53.7	2237 (52.4	11109

n	(33.03%)				%)			4%)	6%)	(34.83%)
Prior CAD	124195 (13.63%)	99246 (13.71%)	11860 (13.08%)	111106 (13.64%)	10324 (4.22%)	1052 (5.73%)	282 (3.65%)	371 (5.82%)	399 (9.36%)	1287 (4.03%)

Supplementary Table 2. Demographics in Cedars Sinai Medical Center test set.

Number of ECGs	46,095
Number of patients	46,095
Number of ECGs with five-year followup	22558 (48.94%)
Five-year cardiovascular mortality	3660 (7.94%)
Age	67.3 (15.5)
Non-Hispanic White	28743 (62.4%)
Hispanic White	3839 (8.3%)
Black or African American	7145 (15.5%)
Asian	3101 (6.7%)
American Indian	113 (0.2%)
Pacific Islander	100 (0.2%)
Other/Unknown	3054 (6.6%)
Female	22611 (49.1%)
Earliest date	2012-01-01
Latest date	2019-12-31

Supplementary table 3: demographics in Columbia Medical Center test set.

Number of ECGs	458455
Number of patients	458455
Number of ECGs with five-year followup	202607 (44.19%)
Five-year cardiovascular mortality	12884 (2.81%)
Age	53.75 (18.71)
White	150484 (32.82%)
Black or African American	59361 (12.95%)
Asian	7647 (1.67%)
Pacific Islander	1305 (0.28%)
American Indian	542 (0.12%)
Other/Unknown Race	239116 (52.16%)
Hispanic or Latino	118778 (25.91%)
Female	213929 (46.66%)

Supplementary Table 4: Performance on different subgroups.

	Sensitivity	Specificity	AUC	Incidence
All	0.756 (0.707-0.804)	0.753 (0.746-0.760)	0.832 (0.810-0.855)	307/31899, 0.962413%
Female	0.763 (0.697-0.838)	0.768 (0.758-0.779)	0.837 (0.804-0.875)	139/14263, 0.974550%
Male	0.750 (0.688-0.816)	0.738 (0.727-0.748)	0.826 (0.796-0.858)	168/17636, 0.952597%
Non-Hispanic White	0.733 (0.664-0.803)	0.759 (0.749-0.769)	0.828 (0.798-0.858)	161/14422, 1.116350%
Hispanic White	0.786 (0.669-0.920)	0.779 (0.757-0.801)	0.870 (0.826-0.927)	42/3515, 1.194879%
Asian	0.816 (0.711-0.929)	0.757 (0.737-0.777)	0.847 (0.797-0.902)	49/3874, 1.264843%
Black	0.714 (0.491-0.967)	0.727 (0.688-0.766)	0.770 (0.648-0.915)	14/1130, 1.238938%
Over 60	0.778 (0.725-0.839)	0.661 (0.649-0.674)	0.799 (0.768-0.832)	207/13016, 1.590350%
Under 60	0.710 (0.622-0.799)	0.837 (0.828-0.846)	0.856 (0.821-0.893)	100/15272, 0.654793%
Normal Sinus	0.707 (0.648-0.764)	0.786 (0.778-0.794)	0.823 (0.796-0.851)	225/27468, 0.819135%
Atrial Fibrillation	0.973 (0.946-1.037)	0.216 (0.180-0.254)	0.757 (0.685-0.838)	37/1445, 2.560554%
1st Degree AV Block	0.824 (0.647-1.022)	0.596 (0.558-0.634)	0.765 (0.660-0.889)	17/1495, 1.137124%
Left Bundle Branch Block	1.000 (1.000-1.000)	0.283 (0.225-0.336)	0.835 (0.761-0.923)	14/612, 2.287582%
Right Bundle Branch Block	0.844 (0.722-0.978)	0.587 (0.552-0.622)	0.778 (0.706-0.861)	32/2077, 1.540684%
Paced	0.935 (0.871-1.031)	0.190 (0.145-0.233)	0.789 (0.710-0.878)	31/763, 4.062910%
Survives first year	0.692 (0.610-0.773)	0.753 (0.746-0.760)	0.800 (0.761-0.840)	120/22822, 0.525808%

Supplementary table 5: Comparison of SEER to models custom-trained for different outcomes. SEER AUROC is the performance of the SEER CV Mortality model in predicting the outcome. Custom-trained AUROC is the performance of a new model with the same architecture as SEER trained specifically for that task.

	CV Mortality	ASCVD	HF	MI	AFib
SEER AUROC	0.795 (0.758-0.833)	0.668 (0.649-0.688)	0.755 (0.743-0.770)	0.673 (0.639-0.708)	0.684 (0.665-0.702)
Custom-trained AUROC	0.795 (0.759-0.834)	0.698 (0.680-0.717)	0.818 (0.807-0.830)	0.684 (0.650-0.717)	0.752 (0.734-0.770)
Spearman correlation between two models	1.000 (1.000-1.000)	0.591 (0.581-0.601)	0.628 (0.619-0.638)	0.540 (0.529-0.551)	0.438 (0.425-0.451)

Supplementary Table 6: Features for random forest models

ECG diagnoses	ECG measurements
NSR	meanventrate
AF	meanprint
JUNCTIONAL	meanqrsdur
AFlutter	meanqtint
EAR	meanqtc
AT	pfrontaxis
SVT	phorizaxis
VT	qrsfrontaxis
PACED	qrshorizaxis
BIGEMINY	tfrontaxis
TRIGEMINY	thorizaxis
WENCKEBACH	
PVC	
PAC	
1AVB	
2AVB	
3AVB	
LBBB	
RBBB	
LAFB	
LPFB	
BIFASCICULAR	
NICD	
LAD	
RAD	
RSA	
LONGQT	
WPW	
LVH	
RVH	
LAE	

RAE	
STEL	
ANTERIOR_INFARCT	
SEPTAL_INFARCT	
LATERAL_INFARCT	
INFERIOR_INFARCT	
POSTERIOR_INFARCT	

Supplementary Table 7: Performance of random forest baselines on the Stanford test set.

	Harrell C-statistic	5-year AUC	Sensitivity	Specificity	Positive Predictive Value	F1 score
XGBoost (11 ECG Parameters)	0.700 (0.671-0.726)	0.705 (0.664-0.746)	0.871 (0.820-0.924)	0.336 (0.325-0.348)	0.032 (0.027-0.037)	0.062 (0.052-0.071)
XGBoost (38 Overread Diagnoses)	0.684 (0.653-0.700)	0.677 (0.643-0.712)	0.996 (0.992-1.005)	0.007 (0.006-0.009)	0.024 (0.021-0.026)	0.046 (0.041-0.051)

Supplementary Table 8: OMOP concept codes for conditions and lab values.

Concept Name	Included concept codes	Excluded concept codes
Myocardial infarction	4329847	314666 (old myocardial infarction)
Ischemic stroke and intracranial hemorrhage	372924, 375557, 376713, 443454, 441874, 439847, 432923	
Sudden cardiac death	4048809, 321042, 442289, 4317150, 4132309	437894 (ventricular fibrillation)
Heart failure	316139	315295 (congestive rhumatic heart failure)
Atrial fibrillation	313217	
Heart block	313791	
Cardiomyopathy	321319	
Peripheral vascular disease	321052	
Aortic stenosis	4189343	
Diabetes	443735, 443767, 192279, 443732, 376065, 443729, 201826, 4225656, 4227210, 435216, 37016355, 4228112, 4224254, 200687, 201254, 201531, 4295011	
HDL cholesterol	3007070	
LDL cholesterol	3028437	
Total cholesterol	3027114	
Systolic blood pressure	4152194	
Diastolic blood pressure	4154790	
Smoking status	43054909, 4275495, 4269997	
